# Exploring Haematological Complications in Cirrhosis of the Liver: A Comprehensive Review

**DOI:** 10.7759/cureus.65319

**Published:** 2024-07-24

**Authors:** Parav Tantia, Parth Aggarwal, Sourya Acharya, Sunil Kumar, Manjeet Kothari, Abhinav Kadam, Rajvardhan Patil

**Affiliations:** 1 Medicine, Jawaharlal Nehru Medical College, Datta Meghe Institute of Higher Education and Research, Wardha, IND

**Keywords:** leukopenia, coagulopathy, thrombocytopenia, anemia, hematological complications, cirrhosis

## Abstract

Various chronic liver diseases inevitably end up with cirrhosis of the liver, and this comes with a whole range of haematological complications. Therefore, this detailed review has discussed pathophysiology, clinical manifestations, diagnostic measures, and treatment plans for these anomalies. Closely related are conditions such as anaemia, thrombocytopenia, coagulopathy, leukopenia, and haemolytic disorders, which are known to contribute to morbidity and mortality in cirrhotic patients significantly. Therefore, we need to understand the causes of these problems to find ways of helping our patients better. For this reason, multidisciplinary management will be key in ensuring proper monitoring, timely intervention, and preventive measures for haematological abnormalities in cirrhosis. Additionally, there have been tremendous advancements in therapeutic options, like adjunctive therapies or haematopoietic growth factors, which hold much promise regarding patient outcomes. This article emphasizes the proactive management of haematological complications associated with cirrhosis while highlighting the need for further research coupled with collaboration aimed at strengthening prevention strategies, diagnostic methods, and curative interventions.

## Introduction and background

Cirrhosis is a chronic liver disease marked by widespread fibrosis and the development of regenerative nodules, which eventually results in impaired liver function. It is the terminal phase of various liver conditions that include persistent hepatitis, alcohol-related liver disease, and non-alcoholic fatty liver disease [1]. Patients with cirrhosis experience haematological complications, which increase morbidity and mortality. These complications encompass a range of disorders: anaemia, thrombocytopenia, coagulopathy, leukopenia, and haemolytic disorders. Appreciating their pathogenesis and clinical implications is important for optimizing patient management and outcomes [2].

This article provides an in-depth analysis of haematological abnormalities associated with liver cirrhosis. By discussing the mechanisms and symptoms experienced, ways to determine them clinically, and ways to manage these complications, this article seeks to give medical professionals a better understanding of what they are dealing with when caring for patients suffering from cirrhotic patients having haematologic abnormalities. Finally, it wants to help improve patient care and outcomes in difficult clinical settings.

## Review

Search methodology

To conduct a comprehensive review on "Exploring Haematological Complications in Cirrhosis of the Liver," a systematic search methodology was implemented. Databases, including PubMed, Medline, and the Cochrane Library, were queried using keywords such as "cirrhosis," "liver disease," "haematological complications," "anaemia," "thrombocytopenia," "leukopenia," and "coagulopathy." Articles published between January 2000 and December 2023 were considered. Inclusion criteria encompassed peer-reviewed articles, clinical trials, observational studies, meta-analyses, and reviews addressing the haematological complications associated with liver cirrhosis in adults. Exclusion criteria included studies focusing solely on paediatric populations, non-English publications, case reports, conference abstracts, and articles not directly related to the haematological aspects of cirrhosis. Only studies with full-text availability were reviewed to ensure comprehensive data analysis. The gathered data were then synthesized to provide an in-depth understanding of the prevalence, mechanisms, clinical manifestations, diagnostic approaches, and management strategies for haematological complications in cirrhosis.

Anaemia in cirrhosis

Prevalence and Types of Anaemia

Anaemia represents a prevalent complication in liver cirrhosis, with studies indicating its occurrence in approximately 66-75% of patients across varying degrees of disease severity [3,4]. Moreover, a substantial proportion of cirrhotic individuals experience severe anaemia, with reported prevalence rates reaching up to 70.2%, and 9.9% presenting with severe anaemia [5]. The predominant form of anaemia observed in liver cirrhosis is normocytic normochromic anaemia, often linked to the chronic inflammatory milieu associated with the condition [4]. Additionally, patients with cirrhosis may exhibit other types of anaemia, including iron-deficiency anaemia, macrocytosis, and deficiencies in vitamin B12 and folate, which can be influenced by factors such as blood loss, malnutrition, and bone marrow suppression [4,6]. The aetiology of anaemia in liver cirrhosis is multifaceted, encompassing acute and chronic blood loss from varices, malnutrition, hypersplenism, and coagulation impairments [4]. Furthermore, alcohol toxicity, vitamin deficiencies, and treatment-related factors, such as medications employed for hepatitis management, may also contribute to the development of anaemia in patients with cirrhosis [4].

Mechanisms of Anaemia in Cirrhosis

The mechanisms underlying anaemia in cirrhosis are multifaceted and intricate, involving a myriad of pathophysiological processes. Chronic inflammation, acute and chronic blood loss from the upper gastrointestinal tract, hypersplenism, haemodilution, and abnormalities in haemostasis all contribute to the development of anaemia in cirrhotic individuals [7-9]. Iron deficiency anaemia (IDA) is a prevalent subtype of anaemia in patients with cirrhosis, particularly in those with early-stage disease, including compensated cirrhosis [9]. Furthermore, the prevalence of anaemia escalates with the progression of cirrhosis severity, with higher incidence rates observed in individuals with decompensated cirrhosis compared to those with compensated cirrhosis [8,9]. The aetiopathogenesis of anaemia in cirrhosis exhibits diversity, with normocytic anaemia often associated with chronic disease, microcytic anaemia attributed to acute blood loss from variceal haemorrhage, and macrocytic anaemia linked to deficiencies in folic acid and vitamin B12 [8]. Moreover, anaemia in cirrhosis represents a significant clinical entity that is frequently underestimated, carrying implications for hepatic decompensation and liver-related mortality [8]. Recognizing the multifaceted nature of anaemia in cirrhosis is crucial for effective management and improved clinical outcomes in affected patients.

Clinical Manifestations and Diagnosis

The aetiology of anaemia in cirrhosis is multifaceted, encompassing chronic inflammation, diminished erythropoietin (EPO) levels, variceal haemorrhage, hypersplenism, medication-related adverse effects, and malnutrition [8,10,11]. Cirrhotic individuals may present with diverse forms of anaemia, including IDA, normocytic anaemia, and macrocytic anaemia, with IDA being particularly prevalent, especially in the early stages of the disease [8,10,11]. Additionally, anaemia in cirrhosis correlates with unfavourable outcomes, such as heightened risks of hepatic decompensation, mortality, and overall poor prognosis [5,10]. Its pathogenesis is often multifactorial, involving factors such as acute or chronic blood loss secondary to portal hypertension, bone marrow suppression due to viral infections, and various other contributory elements [8]. Diagnostic strategies for anaemia in cirrhosis entail elucidating its underlying cause, which may encompass anaemia of chronic disease, acute haemorrhage, and portal hypertensive gastropathy, among other specific triggers [8]. Given its propensity for underdiagnosis, a comprehensive assessment is imperative to discern the specific subtype and aetiology of anaemia in each patient [8]. Effective management of anaemia in cirrhosis necessitates a multifaceted approach, incorporating interventions such as iron, folic acid, and vitamin B complex supplementation; dietary counselling; red blood cell transfusions; vasoconstrictive agents; proton pump inhibitors; and targeted correction of deficiencies such as iron, folate, thiamine, cobalamin, and pyridoxine [8,10,11]. Such comprehensive strategies aim to alleviate symptoms, improve quality of life, and mitigate adverse clinical outcomes in individuals afflicted with cirrhosis-associated anaemia.

Thrombocytopenia and coagulopathy

Overview of Platelet Function and Coagulation in Cirrhosis

Platelet function and coagulation dynamics in cirrhosis exhibit intricate interactions that profoundly influence haemostasis in patients with liver disease. Within cirrhosis, a delicate equilibrium exists between procoagulant and anticoagulant factors, resulting in a state of rebalanced haemostasis [12,13]. This rebalanced hemostasis manifests as a simultaneous reduction in clotting and anticlotting factors, challenging the conventional perception of cirrhosis as solely a hypercoagulable state [13]. Defects in platelet function are commonplace in chronic and acute liver diseases, characterized by impaired platelet-vessel wall interaction and potential proteolytic degradation of platelet receptors, disrupting primary haemostasis [13,14]. Thrombocytopenia, frequently attributed to heightened platelet sequestration in the spleen and diminished thrombopoietin production, is a prevalent characteristic of cirrhosis, substantially impacting platelet counts and function [13,14]. The coagulation dysfunction observed in cirrhosis encompasses various factors, including diminished synthesis of clotting and inhibitor factors, platelet anomalies, hyperfibrinolysis, and augmented intravascular coagulation [13]. Conventional coagulation assays, such as prothrombin time and platelet count, may inadequately gauge bleeding or thrombotic risks in cirrhotic patients, thereby necessitating the adoption of alternative assessments, such as thromboelastography, to afford a more comprehensive evaluation of haemostatic function [13].

Causes and Consequences of Thrombocytopenia

Thrombocytopenia, characterized by a diminished platelet count, can arise from various causes, each with its implications. These causes encompass a spectrum of conditions, including bone marrow disorders such as aplastic anaemia, myelodysplastic syndrome, and leukaemia; infectious agents like HIV triggering immunologic thrombocytopenia; medications such as chlorpropamide; autoimmune diseases like immune thrombocytopenic purpura (ITP); liver diseases such as cirrhosis and hepatitis C virus (HCV); pregnancy-related disorders including preeclampsia and haemolysis; elevated liver enzymes; and sepsis, which can induce nonimmunologic thrombocytopenia via mechanisms like platelet apoptosis and complement activation [15]. The consequences of thrombocytopenia predominantly revolve around heightened bleeding risks. Individuals afflicted with thrombocytopenia may manifest symptoms such as easy bruising, prolonged bleeding from minor cuts, bleeding gums, and heavy menstrual bleeding [16]. The severity of thrombocytopenia spans from mild to severe, with severe thrombocytopenia posing a considerable risk of life-threatening bleeding, particularly when platelet counts plummet below 10,000 platelets per microliter. Severe thrombocytopenia is closely associated with profound internal bleeding, including potentially fatal haemorrhages in critical regions such as the brain. Additionally, thrombocytopenia can precipitate thrombotic complications, encompassing deep venous thrombosis, pulmonary embolism, and arterial thrombosis, notably in scenarios such as heparin-induced thrombocytopenia (HIT) and antiphospholipid antibody syndrome [16].

Coagulation Abnormalities and Bleeding Risk

Cirrhosis precipitates a perturbed equilibrium between pro-coagulant and anti-coagulant factors, heightening the susceptibility to bleeding and thrombotic events [17]. Traditional coagulation assays often fail to accurately depict this intricate coagulation derangement, presenting challenges in evaluating bleeding or thrombotic risks in cirrhotic individuals [12,18]. Variceal bleeding emerges as a prevalent complication in cirrhotic patients, contributing significantly to overall bleeding episodes, characterized by elevated mortality rates and a pronounced risk of recurrent bleeding [17]. Notably, heightened levels of D-dimer and tissue plasminogen activator (t-PA) serve as noteworthy laboratory indicators for variceal bleeding risk, independent of other markers and the severity of liver disease [17]. In cirrhosis, rebalanced haemostasis entails a concurrent reduction in procoagulant and anticoagulant factors, resulting in a delicate equilibrium between thrombosis and bleeding [17,18]. This fragile balance is subject to modulation by factors such as the aetiology and severity of liver disease, acute comorbidities, and ongoing therapeutic interventions, rendering it uniquely tailored to each patient [18]. Standard coagulation tests, including the INR, prothrombin time, and platelet count, often inadequately forecast bleeding risk in cirrhotic patients, and transfusion strategies predicated on these metrics may prove suboptimal for managing coagulopathy in this population [12,18]. Viscoelastic assays, like thromboelastography, have garnered attention as potentially superior tools for gauging the true haemostatic status in cirrhosis, although further validation is warranted to establish transfusion thresholds [19].

Evaluation and Management Approaches

The evaluation and management of liver cirrhosis, particularly in the context of acute oesophageal variceal bleeding, necessitate a multidisciplinary approach aimed at addressing the multifaceted nature of this condition [20]. Contemporary strategies underscore the importance of a thorough assessment and comprehensive treatment plan to effectively navigate the complexities inherent in managing cirrhosis-related complications [20]. The evaluation process typically entails diagnostic tests, including blood work, imaging modalities such as CT scans or MRIs, endoscopic examinations to identify abnormal varices, liver function assessments, and, occasionally, a liver biopsy for detailed histopathological evaluation [21]. These diagnostic interventions play a pivotal role in staging the severity of liver disease and informing treatment decisions. Management strategies for liver cirrhosis complicated by acute oesophageal variceal bleeding commonly entail a multifaceted approach encompassing medical, endoscopic, and interventional therapies. Therapeutic interventions may encompass pharmacological agents to manage fluid retention and toxin accumulation, upper endoscopy for variceal treatment, and advanced procedures such as transjugular intrahepatic portosystemic shunt (TIPS) or balloon-retrograde transvenous obliteration (BRTO) in severe cases [20]. Innovative management approaches for ascites, a prevalent complication of cirrhosis, include dietary sodium restriction, diuretic therapy, and potentially advanced interventions such as TIPS or utilization of an alfapump for refractory cases [22]. These strategies target the underlying pathophysiology of ascites and ultimately enhance patient outcomes and quality of life.

Leukopenia and immunodeficiency

Impaired Immune Function in Cirrhosis

In cirrhosis-associated immune dysfunction (CAID), impaired immune function in cirrhosis represents a multifaceted phenomenon characterized by systemic inflammation and immune paralysis [23]. This dysfunction entails a bidirectional interplay between cirrhosis and immune function, wherein immune-mediated inflammatory mechanisms contribute to the pathogenesis of cirrhosis. In contrast, cirrhosis and portal hypertension induce dysregulated immune cell activation and dysfunction [24]. CAID manifests through two distinct immune phenotypes: a low-grade systemic inflammatory phenotype observed in compensated cirrhosis or decompensation without organ failure, and a high-grade systemic inflammatory phenotype present in acute-on-chronic liver failure, characterized by pronounced immune paralysis and heightened susceptibility to infections, thereby leading to poor prognosis [25]. In cirrhosis, deficiencies in neutrophils and systemic immunity, coupled with portal hypertension-induced splenic sequestration of blood cells, result in numerical and functional deficits in immune cells, particularly neutrophils [23]. The progression of cirrhosis from compensated to decompensated stages and acute-on-chronic liver failure exacerbates systemic inflammation and immune dysfunction, thereby escalating the risk of life-threatening infections [23]. This progression is underscored by compromised immune responses, including impaired phagocytosis, diminished oxidative burst, altered chemotaxis, and reduced production of anti-infective proteins, collectively predisposing individuals to infections and exacerbating clinical outcomes [23]. Comprehending the defective host immunity associated with CAID is pivotal for developing effective diagnostic and therapeutic approaches to address immune dysfunction in cirrhosis patients. Consequently, therapies targeting the modulation of dysfunctional immune responses are undergoing evaluation in both preclinical and clinical settings, intending to attenuate the impact of impaired immune function on cirrhosis progression and patient outcomes [25].

Leukopenia: Incidence, Mechanisms, and Consequences

Leukopenia, characterized by a reduced white blood cell count, can significantly impact an individual's health, with its incidence varying depending on the underlying cause. In solid organ transplant recipients, the occurrence of leukopenia ranges from 7.4% in hepatitis B seropositive kidney and liver transplant recipients [26]. Additionally, leukopenia represents a recognized and potentially perilous adverse effect of myelotoxic therapy, which can impose dose-limiting constraints and pose life-threatening risks [26]. The pathophysiology of leukopenia encompasses four primary mechanisms. Firstly, diminished production of white blood cells may arise due to bone marrow suppression, ineffective granulopoiesis, or genetic abnormalities. Secondly, immune-mediated damage to white blood cells, particularly neutrophils, may occur due to autoimmune disorders or certain medications. Thirdly, augmented peripheral destruction or sequestration of circulating white blood cells constitutes another contributing factor. Lastly, infiltration of the bone marrow by malignant cells, granulomas, or fibrotic tissue can precipitate leukopenia [27]. Medications can induce leukopenia by impeding granulopoiesis (e.g., mycophenolate, calcineurin inhibitors) or augmenting peripheral destruction (e.g., rituximab, antithymocyte globulin) [27]. The most dreaded complication of leukopenia is neutropenia, which elevates the risk of bacterial infections, particularly when the absolute neutrophil count dips below 500 cells/mm³. Patients afflicted with severe neutropenia may present with fatigue, fever, malaise, and oral ulcerations, with a dearth of localizing symptoms due to the scarcity of circulating neutrophils. In sepsis, leukopenia can serve as a dire prognostic indicator, necessitating prompt diagnosis and intervention [28]. In solid organ transplant recipients, leukopenia heightens susceptibility to infections and other complications [28].

Infections in Cirrhotic Patients

Infections represent a pressing concern in cirrhotic patients, given their compromised immune status, rendering them more susceptible to various bacterial infections. Among the common infections encountered in cirrhotic individuals are spontaneous bacterial peritonitis (SBP), urinary tract infections (UTIs), pneumonia, bacteraemia, and cellulitis [29-33]. These infections can precipitate severe consequences and are associated with heightened morbidity and mortality rates in cirrhotic patients [30-32]. Notably, the prevalence of infections in cirrhotic individuals surpasses that of the general population, particularly among those with gastrointestinal bleeding [30]. Gram-negative bacteria, including *Escherichia coli*, *Klebsiella* spp., and *Enterobacter* spp., emerge as primary causative agents. However, gram-positive bacteria such as *Enterococci* and *Staphylococcus aureus* are also implicated in infections among cirrhotic patients [30]. Infections in cirrhotic individuals may stem from either community-acquired or nosocomial sources, with nosocomial infections often exhibiting higher mortality rates and resistance to antibiotics [30-32]. The prevalence of multidrug-resistant microorganisms in cirrhotic patients poses significant challenges in empirical antimicrobial therapy, necessitating vigilant surveillance and implementation of appropriate management strategies to mitigate mortality rates associated with sepsis in this population [32].

Haemolytic disorders in cirrhosis

Overview of Haemolytic Disorders Associated With Cirrhosis

Haemolytic disorders represent a frequent occurrence in individuals afflicted with cirrhosis of the liver, posing a spectrum of challenges and complications. One noteworthy variant is spur cell haemolytic anaemia, distinguished by the presence of acanthocytes or spur cells that undergo splenic entrapment followed by haemolysis. This condition is intimately intertwined with severe liver dysfunction and bears an ominous prognosis, often necessitating urgent assessment for liver transplantation to address the swiftly deteriorating anaemia. Additionally, Zieve syndrome, an uncommon entity encompassing haemolytic anaemia, cholestatic jaundice, and hyperlipidaemia, can manifest within the context of alcoholic liver disease. Treatment primarily revolves around alcohol abstinence, with most patients exhibiting improvement within a few weeks [34]. Cirrhosis associated with chronic HCV infection may also precipitate Coombs-negative autoimmune haemolytic anaemia (AIHA). This medically manageable entity might be overlooked as a contributor to anaemia in HCV liver disease. Despite yielding a negative direct Coombs test result, detecting heightened red blood cell-bound IgG antibodies underscores the significance of autoimmune haemolysis in these patients. Furthermore, other haemolytic disorders, such as burr cell haemolytic anaemia and haemolysis secondary to severe liver dysfunction stemming from various aetiologies, have been documented in the milieu of cirrhosis, further complicating the landscape of haematological complications in these individuals [35].

Mechanisms and Clinical Manifestations

Liver disease, particularly alcoholic cirrhosis, instigates chemical alterations that precipitate structural and metabolic aberrations in the erythrocyte membrane, culminating in haemolytic anaemia. These changes encompass shifts in the cholesterol-phospholipid ratio, expansion of the lipid bilayer, and modifications to the erythrocyte surface induced by abnormalities in high-density lipoprotein (HDL) [36]. Haemolysis in liver disease can manifest as either intrinsic, stemming from erythrocyte membrane abnormalities, or extrinsic, influenced by environmental factors. While intrinsic causes are predominantly hereditary, extrinsic factors encompass immune and nonimmune mechanisms. Liver disorders, such as alcohol-induced liver damage, can induce severe hypophosphatemia, exacerbating red cell membrane fragility and spheroidicity, rendering red cells more susceptible to splenic trapping and subsequent haemolysis [37]. Haemolytic anaemia in liver disease typically manifests with rapid-onset anaemia, jaundice, a history of pigmented gallstones, splenomegaly, and mild hepatomegaly. Laboratory assessments reveal elevated serum lactate dehydrogenase (LDH) levels, mildly increased serum aspartate transaminase (AST) levels, and heightened total bilirubin levels.

**Figure 1:**
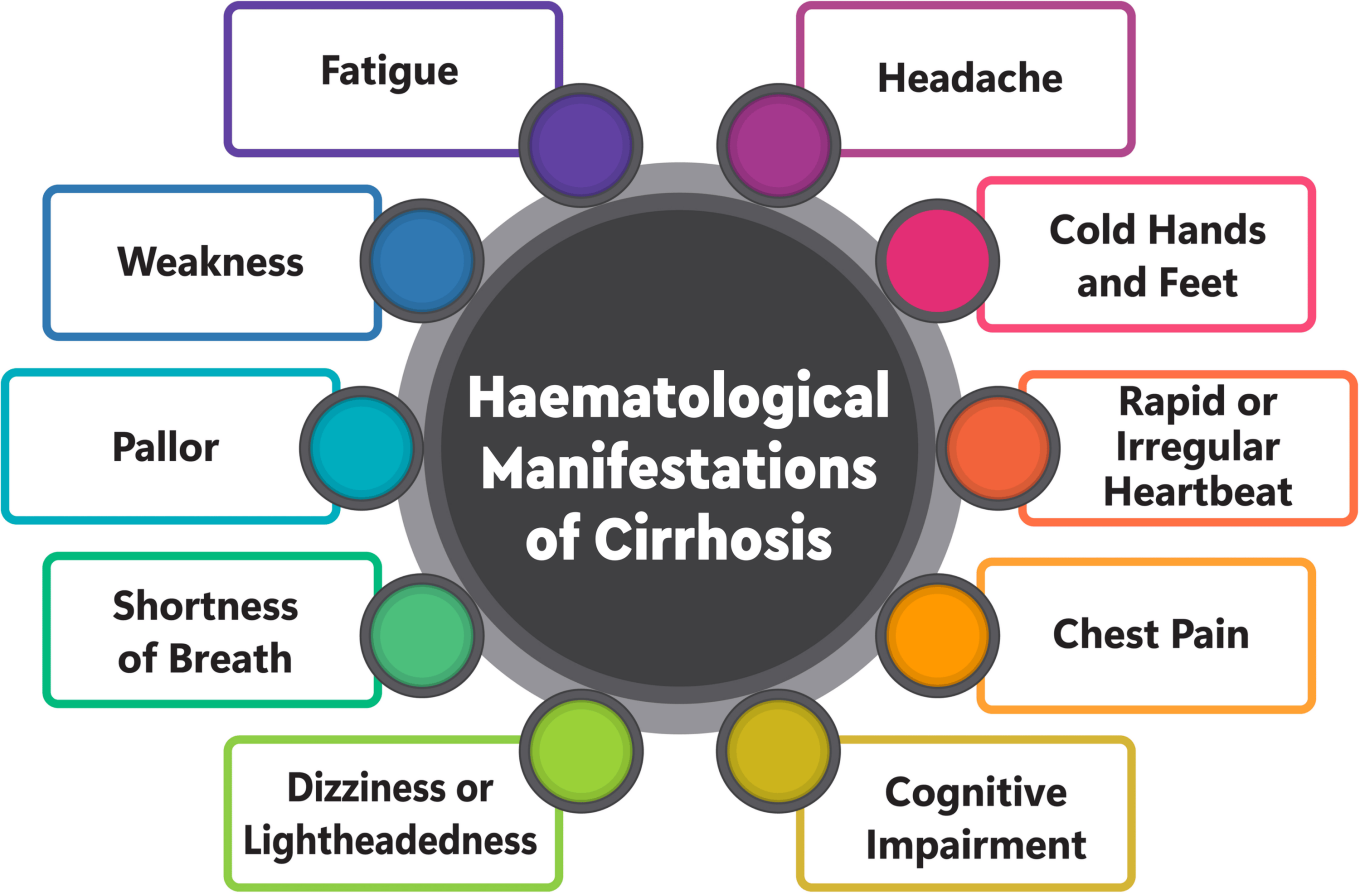
Clinical manifestations of anaemia Image credit: Dr. Parav Tantia

Moreover, liver dysfunction can be precipitated by blood transfusions in conditions such as sickle cell disease and thalassemia [38]. Spur cell haemolytic anaemia, characterized by acanthocytes or spur cells, represents a severe manifestation of haemolysis linked to liver dysfunction. These morphologically altered red blood cells undergo splenic entrapment, culminating in extravascular haemolysis and ensuing anaemia. Despite often being overlooked, spur cell anaemia carries an unfavourable prognosis, underscoring the imperative of early diagnosis and prompt management in cirrhosis patients (Figure 1) [39].

Diagnosis and Treatment Considerations

The diagnosis and treatment considerations for haemolytic disorders in cirrhosis represent critical components of managing these intricate conditions. In cirrhosis patients, spur cell haemolytic anaemia can present a formidable challenge, characterized by acanthocytes in the blood smear. A meticulous evaluation, inclusive of peripheral smear analysis, holds paramount importance in diagnosing alternate and potentially fatal causes of anaemia, such as spur cell anaemia, in cirrhosis patients. Healthcare providers must remain vigilant in identifying and distinguishing haemolytic disorders to initiate appropriate treatment and avert complications promptly [40].

AIHA can also manifest in cirrhosis patients due to diverse factors, encompassing infections, medications, and autoimmune conditions. Considering autoimmune aetiologies of haemolysis in cirrhosis patients presenting with anaemia is imperative. Conducting pertinent diagnostic tests to elucidate the underlying cause of haemolysis is indispensable for effective management. Treatment strategies pivot on addressing the root cause of haemolytic anaemia in cirrhosis patients, with prompt intervention serving as a linchpin in thwarting disease progression and enhancing patient outcomes [41].

In instances of severe haemolytic anaemia in cirrhosis, particularly spur cell anaemia, patients may necessitate urgent assessment for liver transplantation [42]. Early diagnosis and management are paramount in averting complications and bolstering prognosis in these critical scenarios. Additionally, in conditions such as Zieve syndrome, which is characterized by haemolytic anaemia in the backdrop of alcoholic liver disease, abstinence from alcohol assumes a pivotal role in management. Recognizing specific syndromes and their ramifications can steer appropriate interventions, obviate unnecessary procedures, and foster recovery in patients grappling with alcohol-related haemolysis. Overall, adopting a comprehensive approach encompassing accurate diagnosis, addressing underlying causes, contemplating transplant evaluation when warranted, and advocating lifestyle modifications is indispensable for effectively managing haemolytic disorders in cirrhosis and ameliorating patient outcomes [42].

Role of haematopoietic growth factors

EPO and Its Role in Managing Anaemia

EPO is primarily synthesized by the kidneys, orchestrating the stimulation of red blood cell production, pivotal for oxygen delivery to bodily tissues [43,44]. Diminished levels of EPO can precipitate anaemia, characterized by either a diminished count of red blood cells or impaired red blood cell functionality [44]. Recombinant erythropoietin (rHuEPO) has heralded a transformative era in anaemia management, particularly among chronic renal failure patients, engendering increased survival rates, reduced hospitalizations, enhanced cognitive function, and elevated quality of life [44]. It has emerged as an indispensable adjunct in addressing anaemia-concomitant chronic maladies.

In congestive heart failure (CHF), anaemia represents a prevalent finding linked with escalated mortality rates. Erythropoiesis-stimulating agents (ESAs), including EPO, have been deployed in managing anaemia among CHF patients, albeit recent investigations have yielded conflicting outcomes concerning their efficacy [45]. The utilization of ESAs in anaemic individuals with heart failure remains a subject of contention. EPO therapy is paramount in conditions marked by endogenous EPO production deficiency, such as chronic kidney disease, fostering red blood cell production stimulation and alleviating anaemia burdens [44]. Its role in maintaining optimal haemoglobin levels and ameliorating overall patient outcomes cannot be overstated.

Thrombopoietin in Treating Thrombocytopenia

In ITP, thrombopoietin receptor agonists (TPO-RAs) such as romiplostim and eltrombopag exhibit substantial enhancements in platelet response rates and durable responses compared to placebo [46-48]. They additionally mitigate the incidence of bleeding events and diminish the necessity for rescue medications [46,47]. TPO-RAs afford favourable prospects for maintaining platelet counts through daily or weekly treatment regimens, enabling patients to undergo complete courses of antiviral therapy for HCV [47]. This proves pivotal in achieving a sustained virologic response (SVR) in cirrhosis patients grappling with thrombocytopenia. In a study involving chronic ITP patients, eltrombopag demonstrated efficacy in elevating platelet counts and mitigating bleeding episodes during treatment [47]. Notably, TPO-RAs boast a commendable safety profile, exhibiting akin rates of adverse events compared to placebo [46,47]. Eltrombopag has further demonstrated successful application as frontline therapy in conjunction with high-dose dexamethasone for newly diagnosed adult primary ITP [48]. This combined therapeutic approach showcased superior response rates compared to dexamethasone monotherapy.

Potential Benefits and Limitations of Growth Factor Therapy

Haematopoietic growth factors emerge as pivotal components in the management of haematological complications intertwined with liver cirrhosis. These complications, spanning anaemia, leukopenia, and thrombocytopenia, represent prevalent occurrences among cirrhosis patients, exerting profound impacts on their prognoses. rHuEPO and granulocyte colony-stimulating factor (G-CSF) stand as stalwart agents in addressing these cytopenias, fostering enhanced tolerability and adherence to antiviral therapy in cirrhosis patients. Studies underscore the efficacy of growth factors like eltrombopag, a TPO-RA, in augmenting platelet counts, facilitating a more significant proportion of patients to commence and sustain interferon-based therapy, ultimately bolstering treatment outcomes [49]. In hepatology, haematopoietic growth factors wield particular significance for cirrhosis patients embarking on antiviral therapy, affording optimal dosing of medications pivotal for attaining SVR. These growth factors mitigate the adverse haematological ramifications of interferon and ribavirin and contribute to liver regeneration and immunopathogenesis. Stem cell factor (SCF), epidermal growth factor (EGF), and angiopoietin-2 stand among the growth factors implicated in liver fibrosis and cirrhosis, serving as indices of disease progression and prospective therapeutic targets [50]. While growth factor therapy proffers substantive advantages in managing haematological complications and optimizing antiviral treatment in cirrhosis patients, attendant limitations warrant consideration. These constraints encompass the short in vivo half-life of growth factors, the substantial protein requisite for local administration, intricate dosage deliberations, concerns regarding tolerability, and the conceivable emergence of resistance. Notwithstanding these challenges, the therapeutic promise of haematopoietic growth factors in cirrhosis remains auspicious, accentuating the imperative of sustained research endeavours and clinical implementation to ameliorate patient outcomes within this cohort [51]. Table 1 shows the summary of the article in the review.

**Table 1:** List of included studies in the review HCV: Hepatitis C virus

Author	Year	Details and elaboration of findings
Bajaj et al. [1]	2011	This study explores the extensive burden of cirrhosis and hepatic encephalopathy on patients and their caregivers, highlighting the multifaceted impacts on quality of life.
Sharma and John [2]	2023	A comprehensive overview of hepatic cirrhosis, discussing its aetiology, pathophysiology, clinical manifestations, and the broad spectrum of complications including haematological.
Scheiner et al. [3]	2020	Investigates the prevalence and risk factors of anaemia in patients with advanced chronic liver disease, emphasizing the need for targeted diagnostic and therapeutic approaches.
Singh et al. [4]	2020	Examines the correlation between the severity of liver cirrhosis and anaemia, providing insights into the clinical implications and management strategies for affected patients.
Dawidowski and Pietrzak [6]	2022	Discusses rare causes of anaemia in liver diseases, highlighting less common aetiologies that clinicians should consider in differential diagnosis and treatment plans.
Marginean et al. [7]	2023	Provides an overview of the diagnostic approaches and pathophysiological mechanisms of anaemia in chronic liver disease, offering a framework for understanding and managing these cases.
Manrai et al. [8]	2022	Highlights anaemia as an often underestimated complication in cirrhosis, advocating for more awareness and comprehensive management in clinical practice.
Singh et al. [12]	2022	Discusses the concept of rebalanced haemostasis in cirrhotic coagulopathy, offering insights into the changes in haemostatic mechanisms and their clinical relevance.
Islam et al. [13]	2022	Examines the mechanisms of haemostasis changes in liver failure and their management, providing practical guidance for managing coagulopathy in cirrhosis patients.
Lisman and Porte [14]	2012	Focuses on platelet function in cirrhosis, highlighting how cirrhosis affects platelet production and function, and the implications for bleeding and thrombotic complications.
Jinna and Khandhar [15]	2024	Reviews thrombocytopenia, detailing its causes, clinical presentation, and management, particularly in the context of liver diseases like cirrhosis.
Kucukyurt and Eskazan [16]	2020	Discusses the assessment and monitoring strategies for patients with immune-mediated thrombotic thrombocytopenic purpura (iTTP), aiming to improve patient outcomes.
Muciño-Bermejo et al. [17]	2013	Reviews coagulation abnormalities in cirrhotic patients, emphasizing the need for careful management of bleeding risks and coagulopathy.
Li et al. [18]	2018	Investigates the association of coagulopathy with bleeding risks after invasive procedures in cirrhosis, providing guidelines for safer procedural interventions.
Rautou et al. [19]	2023	Reviews bleeding and thrombotic complications in cirrhosis, offering a comprehensive appraisal of the current state-of-the-art in diagnosis and management.
Garbuzenko [20]	2016	Discusses current approaches to managing acute oesophageal variceal bleeding in cirrhosis patients, highlighting therapeutic strategies and outcomes.
Wong [22]	2023	Reviews innovative approaches to managing ascites in cirrhosis, emphasizing novel therapeutic interventions and their impact on patient outcomes.
Hasa et al. [23]	2022	Explores the link between liver cirrhosis and immune dysfunction, detailing how cirrhosis impacts immune responses and the implications for infection management.
Liaskou et al. [24]	2019	Provides insights into cirrhosis-associated immune dysfunction, particularly focusing on impaired adaptive immunity and potential therapeutic targets.
Albillos et al. [25]	2022	Reviews cirrhosis-associated immune dysfunction, discussing novel insights and therapeutic approaches to manage immune-related complications in cirrhosis patients.
Hartmann et al. [26]	2008	Discusses management strategies for leukopenia in kidney and pancreas transplant recipients, with relevant implications for cirrhosis patients experiencing leukopenia.
Mart et al. [27]	2022	Investigates the aetiology of isolated leukopenia in patients, providing diagnostic and management guidelines applicable to cirrhosis-related leukopenia.
Newburger and Dale [28]	2013	Reviews evaluation and management of patients with isolated neutropenia, offering insights into approaches relevant to cirrhosis-related neutropenia.
Lameirão Gomes et al. [29]	2018	Discusses bacterial infections in cirrhosis patients, emphasizing practical guidelines for prevention and treatment in internal medicine settings.
Bunchorntavakul et al. [30]	2016	Critically reviews bacterial infections in cirrhosis, providing practical guidance for clinicians to manage these infections effectively.
Bajaj et al. [31]	2021	Discusses the evolving challenge of infections in cirrhosis, highlighting new insights and therapeutic strategies for managing infection risks in these patients.
Miranda-Zazueta et al. [32]	2020	Reviews current treatment strategies for bacterial infections in cirrhosis, emphasizing the importance of timely and effective antimicrobial therapy.
Fernández et al. [33]	2021	Reviews management of bacterial and fungal infections in cirrhosis, focusing on the challenge posed by multi-drug resistant organisms (MDROs).
Miwa et al. [34]	2020	Case report and literature review on spur cell anaemia related to alcoholic liver cirrhosis managed without liver transplantation.
Basseri et al. [35]	2010	Case report and review on autoimmune haemolytic anaemia in treatment-naive chronic hepatitis C infection.
Morse [36]	1990	Reviews the mechanisms of haemolysis in liver disease, providing a foundational understanding applicable to various haematological complications in cirrhosis.
Murakami and Shimizu [37]	2013	Reviews hepatic manifestations in haematological disorders, offering insights into how haematological conditions can impact liver function and vice versa.
Baldwin et al. [38]	2024	Comprehensive review on haemolytic anaemia, detailing current knowledge and perspectives relevant to its occurrence in liver disease contexts.
Shah et al. [39]	2024	Reviews acanthocytosis, discussing its relevance to liver cirrhosis and the haematological complications associated with it.
Bevilacqua et al. [40]	2023	Identified spur cells as predictors of acute-on-chronic liver failure and liver-related mortality, independent of severe anaemia.
Michalak et al. [41]	2020	Reviewed current knowledge and perspectives on autoimmune haemolytic anaemia, highlighting mechanisms and treatment options.
Kedarisetty and Kumar [42]	2020	Discussed severe and transfusion-refractory anaemia caused by spur cells in patients with acute-on-chronic liver failure.
Tong and Nissenson [44]	2001	Explored the role of erythropoietin in managing anaemia, particularly in patients with kidney disease.
Palazzuoli et al. [45]	2014	Investigated the use of erythropoietin-stimulating agents in anaemic patients with heart failure, discussing benefits and challenges.
Wang et al. [46]	2016	Conducted a systematic review and meta-analysis on the efficacy and safety of thrombopoietin receptor agonists in primary immune thrombocytopenia.
Kuter [47]	2021	Focused on the treatment of immune thrombocytopenia, with an emphasis on thrombopoietin receptor agonists.
Pulanić et al. [48]	2023	Provided a systematic review and expert consensus on the use of thrombopoietin receptor agonists in adults with immune thrombocytopenia.
Qamar and Grace [49]	2009	Examined abnormal haematological indices in cirrhosis, discussing various blood disorders associated with the condition.
Mancino et al. [50]	2010	Discussed the use of haematopoietic growth factors to manage haematological side effects of antiviral treatment in HCV hepatitis.
Chung et al. [51]	2014	Evaluated whether antiviral therapy reduces complications of cirrhosis, focusing on clinical outcomes and liver function.

Limitations

Despite providing a thorough overview of the haematological complications associated with liver cirrhosis, this review article has certain limitations. The scope is restricted by the variability in clinical manifestations and the multifactorial nature of these complications, which makes it challenging to draw universally applicable conclusions. Additionally, the reliance on existing literature means that the review is limited by the quality and breadth of available studies, which may not cover all aspects of these complex interactions. The heterogeneity of patient populations and differing diagnostic criteria across studies further complicate the synthesis of data. Future research should focus on large-scale, multicentric studies and standardized diagnostic protocols to better understand and manage these haematological complications. 

## Conclusions

In conclusion, exploring the haematological complications in cirrhosis of the liver reveals a complex interplay of factors contributing to conditions such as anaemia, thrombocytopenia, coagulopathy, leukopenia, and haemolytic disorders. These blood-related issues significantly impact patient morbidity and mortality, necessitating a comprehensive approach to diagnosis and management. Early detection, targeted treatment strategies, and ongoing research into the underlying mechanisms are crucial for improving patient outcomes. The integration of multidisciplinary care, along with advances in therapeutic interventions, offers hope for better managing these complications and enhancing the quality of life for patients with cirrhosis.
